# Sexual Behaviour, HIV Prevention and Testing Among Transgender and Non-binary People in a Cross-Sectional Behavioural Surveillance Survey in Aotearoa New Zealand

**DOI:** 10.1007/s10461-026-05279-z

**Published:** 2026-08-11

**Authors:** Peter J.W. Saxton, Ryan M. Bentham, Brooke M. Hollingshead, Jack Byrne, Ashe Yee, Janine Paynter, Koson Tony Sriamporn

**Affiliations:** 1 School of Population Health, Faculty of Medical and Health Sciences, University of Auckland, Auckland, New Zealand; 2 Transgender Health Research Lab, School of Psychology, University of Waikato, Hamilton, New Zealand

**Keywords:** Transgender, Non-binary, Disaggregation, Surveillance, HIV, Sexual health

## Abstract

Trans and non-binary (TNB) people who have sex with gay, bisexual and other men who have sex with men (GBM) are rarely included in HIV behavioural surveillance. Improved data on risk and protective behaviours among TNB people could help identify unmet needs. We analysed data from the Sex and Prevention of Transmission Study, a cross-sectional HIV behavioural surveillance programme in Aotearoa New Zealand that extended eligibility to TNB participants for the first time in 2022. We categorised TNB participants who reported sex with GBM < 5 years into four groups: trans women (TW), trans men (TM), non-binary people assigned male at birth (AMAB), non-binary people assigned female at birth (AFAB). TNB participants were younger, more socioeconomically disadvantaged, and reported more long‑term disability than cis GBM. Compared to cis GBM, TM and non-binary AFAB participants reported fewer male partners and more sex with women and non‑binary partners, whereas TW and non-binary AMAB participants’ partnering more closely resembled cis GBM. HIV testing < 12 months was lower across all TNB groups, and PrEP < 6 months was lower among TM and non-binary AFAB participants. Sexually transmitted infection screening and diagnosis < 12 months appeared similar across all participants; few TNB participants reported living with HIV. TNB participants reported higher levels of negative healthcare experiences and injecting drug use, but not methamphetamine use. Each TNB subpopulation had a unique behavioural profile that differed from both other TNB participants and cis GBM. Lower uptake of HIV testing and PrEP could reflect less risk exposure, service barriers, or both.

## Background

Since the start of the AIDS epidemic, considerable research has focused on gay, bisexual and other men who have sex with men (GBM) who have been disproportionately affected by HIV [1]. Within this literature, studies have often ignored gender diversity. Transgender women have been grouped, misclassified or conflated with GBM, masking differences in HIV prevalence estimates [2]. Transgender men and non-binary people were likely included in GBM samples historically, but without measures of sex assigned at birth and gender identity, remain unrecognised. The erasure of trans identity in HIV epidemiological and behavioural research has also been described as a barrier to pre-exposure prophylaxis (PrEP) uptake for trans women [3]. Moreover, the absence of HIV data on trans men and non-binary people, including those who have sex with GBM, is only slowly and inconsistently starting to be addressed. For example, a recent meta-analysis estimated the global prevalence of HIV in transgender people as 21% [4], highlighting the disproportionate impacts on trans women, particularly in Asia and Africa, and also on trans men in Asia. Likewise, an Australian cohort study has shown HIV incidence among transgender people to be higher than among cis men and women, and similar to cis gay and bisexual men [5].

HIV behavioural surveillance systems – regular cross-sectional sampling of key populations such as GBM – emerged in the 2000's to monitor progress on HIV prevention and testing uptake, identify inequities and target interventions [6, 7]. As with HIV epidemiological surveillance, HIV behavioural surveillance systems have a mixed record on trans inclusion and visibility. Differences in approaches can arise from conceptual, behavioural, social, healthcare and epidemiological complexities. Conceptually, sexual identity, sexual practices, gender identity and sexual partnering are distinct dimensions that nevertheless overlap [8–10]. Behaviourally, trans and GBM populations exhibit diverse sexual repertoires [11], but public health attention has often focused exclusively on anal intercourse that carries greater biological per-contact HIV transmission risk [12]. Socially, GBM, trans and non-binary individuals often interact in similar physical and online spaces, having implications for sampling strategies and study promotion [13]. Trans and GBM populations collectively report poor experiences with healthcare systems, but each population may have unique sexual health risks, needs and priorities [14]. Epidemiologically, trans women, trans men and non-binary individuals may also have different sexual mixing propensities with GBM [15–19], in whom HIV is most concentrated, influencing their probability of HIV exposure.

In response to such challenges, national HIV behavioural surveillance programmes have begun reviewing and adapting their study protocols [13, 20–25]. Australian researchers redesigned a longstanding system to be more inclusive of trans men, non-binary and bisexual participants [13]. Changes included adopting gender-neutral language, and expanding questions on non-anal sexual behaviours, female and non-binary partners, and a broader range of relationship types [13]. However, few national programmes have reported on the sexual behaviours, HIV risk-reduction practices or testing uptake of TNB participants newly included in behavioural surveillance, or examined how these patterns compare to GBM typically sampled [25]. Doing so could help identify HIV and STI prevention inequities for TNB populations and guide targeted public health action. It may also support those conducting behavioural surveillance to determine how best to summarise findings for research end-users, including decisions about data disaggregation and/or analytic adjustments.

Despite the growing recognition of TNB individuals within behavioural surveillance systems, there is limited research on the sexual health of TNB people in Aotearoa New Zealand (hereafter, “NZ”) [26–28]. NZ is among a small number of countries to have estimated the TNB population in its national census [29–31] comprising 0.7% of the population aged 15 and over in 2023 [29]. NZ also has a distinct cultural profile of TNB individuals, encompassing Western, non-Western, and indigenous identities, such as Māori takatāpui and whakawāhine, Rarotongan akava’ine, and Samoan fa’afafine [32]. NZ’s epidemiological surveillance indicates that a low proportion of individuals ever diagnosed with HIV are trans (0.8%, or 51 out of 6452) [33], however, this may underestimate the true count due to historic coding practices in notification forms. Furthermore, the absence of behavioural data hinders the interpretation of epidemiological data. Collectively, unmet prevention needs for this understudied population are consequently difficult to assess.

Our paper addresses this gap by examining data from NZ’s HIV behavioural surveillance programme that was adapted to include TNB populations. We describe the socio-demographic characteristics of TNB participants, analyse their HIV testing, risk and protective behaviours, and examine whether these differed from cis GBM sampled that year. Based on these findings, we aim to make recommendations for future behavioural surveillance in NZ and other countries.

## Methods

We analysed data from the 2022 round of the Sex and Prevention of Transmission Study (SPOTS), a repeat cross-sectional survey that comprises NZ’s national HIV behavioural surveillance programme [34]. From 2002 to 2014, eligible participants were cisgender men aged 16 years and older, who were living in NZ and had sex with another man in the previous six months. In 2022, eligibility was widened to include those who met any of the following: men (cis or trans) who had ever had sex with a man; men (cis or trans) who had not had sex with a man but identified as gay, bisexual, pansexual, queer or takatāpui (an indigenous Māori term that includes someone attracted to the same gender); trans women and non-binary individuals who had had sex with GBM in the previous five years. These criteria were chosen to reflect NZ’s epidemiological profile, where local transmission is concentrated among GBM [33], whilst opening participation to trans and non-binary individuals more likely to be exposed to HIV. Notably, this behavioural surveillance system is explicitly focused on trans participants who are sexual partners of GBM, and is therefore not inclusive of, nor representative of, all trans populations.

The questionnaire included items on sexual behaviours, HIV/STI prevention and testing, healthcare engagement, and attitudes and knowledge. To improve the inclusivity of the survey’s language and the appropriateness of response options for non-cisgender GBM, the team consulted with trans community and academic stakeholders and made changes. Participation was self-completed, online, anonymous and voluntary. A marketing campaign publicised the study across mainstream media channels, community organisations, strategic locations such as sexual health clinics and gay bars, and online spaces including social networking sites and dating apps. A subset of the 2022 round’s marketing collateral was designed to appeal directly to trans individuals, informed by trans community peers, and the study eligibility criteria were clearly displayed on the survey web portal and participant information sheet. Electronic informed consent was required to proceed to the questionnaire. The study was funded by the NZ Ministry of Health and Health Research Council of NZ. Ethics approval was received from NZ’s Health and Disability Ethics Committee (HDEC 2021 11450). Data were collected from 26 April to 4 August 2022.

### Measures

Except for gender, we dichotomised any responses with more than two response options due to the low numbers of trans participants, compared to cis GBM.

### Gender

Trans people, or people whose gender is different from their sex assigned at birth, include those with a binary gender (i.e., trans women/women or trans men/men) and those with a non-binary gender (any gender other than solely woman or man) [32]. At the start of our survey, participants were asked a set of compulsory questions to determine eligibility, including their gender, the sex recorded on their original birth certificate (sex assigned at birth), if they had had sex with a man ever, or in the five years prior to survey, as well as a multi-response item on sexual, gender or cultural identities. For participants who had not yet had sex with a man, we confirmed their sexual, gender or cultural identity was gay, bisexual, pansexual, queer, or takatāpui. Using these responses, we grouped participants into the following five categories: [32]

- Trans women (TW) included those whose gender was ‘woman’, whose sex assigned at birth was ‘male’, and who indicated they had engaged in sex with GBM in the last five years;
- Trans men (TM) included those whose gender was ‘man’ and whose sex assigned at birth was ‘female’, and who either indicated they had engaged in sex with GBM in the last five years, or identified as gay, bisexual, pansexual, queer, or takatāpui;
- Non-binary participants assigned male at birth (AMAB) included those whose gender was ‘non-binary’, whose sex assigned at birth was ‘male’, and who either indicated they had engaged in sex with GBM in the last five years, or identified as gay, bisexual, pansexual, queer, or takatāpui;
- Non-binary participants assigned female at birth (AFAB) include those whose gender was ‘non-binary’ and whose sex assigned at birth was ‘female’ or ‘prefer not to say’, and who indicated they had engaged in sex with GBM in the last five years;
- Cisgender gay and bisexual men (cis GBM), including all other participants whose gender was ‘man’ and whose sex assigned at birth was ‘male’, who had either had sex with a man at least once in their life, or who identified as gay, bisexual, pansexual, queer, or takatāpui.

We refer to these categories as groups throughout the paper. Where applicable, we report results for all non‑binary participants combined (inclusive of AMAB and AFAB). Where data are reported for all TNB participants, this is inclusive of TM, TW, non-binary participants AMAB and non-binary participants AFAB.

### Sexual Partnering

We asked participants when they last had sex with a man (cis or trans) and dichotomised responses into those who had sex with a man in the last six months and those who had not, including those who had never had sex with a man. We asked participants when they had last had sex with a woman or a non-binary person and dichotomised responses in the same way. For participants who reported sex with men in the last six months, we asked how many (response options 1, 2–5, 6–10, 11–20, 21–50 and more than 50); we then dichotomised responses into those who had sex with two or more men and those who had sex with one man or none. We also asked participants if they currently had a regular male partner.

### Prevention Behaviours

To determine prevention behaviours during casual sex, we asked participants if they had casual sex with men in the last six months and for those who had, if this included anal intercourse (receptive/bottomed or insertive/topped) and the frequency of condom use (response options ‘always’, ‘almost always’, ‘about half the time’, ‘rarely’ or ‘never’). We dichotomised participants into those who had condomless anal intercourse at least once with a casual partner during the last six months, and those who had consistently used condoms during anal intercourse, had not had anal intercourse with a casual partner, or had not had sex with a man.

### PrEP Use

We asked non-HIV positive participants two questions about PrEP use. Firstly ‘in the last 6 months, did you take PrEP to protect yourself from HIV?’, and secondly ‘New forms of PrEP are currently under development. If all these options were available, equally effective at preventing HIV, and had a similar cost, which if any would you be willing to use in future? (tick all that apply)’. We grouped participants into those who responded with ‘Daily oral pill’ or ‘Two oral pills before sex then one pill each day until two days after sex (On-demand/2-1-1/event-driven PrEP)’ and those who only responded with ‘A long-acting injection from your doctor or nurse every 2 months’ or ‘None of these’.

### Combination HIV Prevention Coverage

We defined participants as having used combination HIV prevention coverage with casual partners in the last six months if they avoided sex or anal intercourse with casual male partners, were using PrEP, were living with diagnosed HIV and had an undetectable HIV viral load, or used condoms consistently [35].

### HIV Testing and Status

We asked participants the result of their last HIV test and dichotomised participants into those who responded HIV positive versus those who responded HIV negative or that they did not know. We then asked participants who did not report a positive diagnosis how many HIV tests they had in the last 12 months and dichotomised participants into those who had no test and those who had one or more.

### Sexual Health Checkups and STI Diagnoses

We asked participants how many sexual health tests they had in the last 12 months. We also asked participants if they had been diagnosed with an STI in the last 12 months. Response options included ‘gonorrhoea’, ‘chlamydia’, ‘syphilis’, ‘non-specific urethritis’, ‘anal or genital warts’, ‘herpes (HSV)’, and ‘no, none of these’. For both questions, we dichotomised participants into those who had none and those who had one or more of these tests/diagnoses.

### Healthcare Experiences

Participants were asked about their experiences with healthcare providers, and specifically, if their general practitioner knew about their sexuality (i.e., gay/bisexual/takatāpui/pansexual/queer) or that they have sex with men. Participants were also asked if they had ever had any negative experiences with a healthcare provider because of their sexuality or sexual health needs.

### Substance Use

We asked participants if they had used methamphetamines in the last six months (a PrEP indication) or if they had ever injected illegal drugs (an HIV risk behaviour).

### Sexual Safety Attitude

Participants were asked how much they agreed with the statement ‘The sex I have is always as safe as I want it to be’ and dichotomised participants into those who ‘agreed’ or ‘strongly agreed’ and those who ‘disagreed’ or ‘strongly disagreed’.

### Knowledge About HIV Treatments and PrEP

We asked participants if they knew, before taking the survey, whether the following two statements were true. The first was ‘Being on HIV treatments, reaching and maintaining an undetectable viral load (UVL) means that someone living with HIV cannot pass on HIV to their sexual partners’. The second statement was ‘PrEP (Pre-Exposure Prophylaxis) is a pill that can be used by someone who is HIV-negative to significantly decrease their risk of acquiring HIV, if taken as prescribed’. For each, we dichotomised participants into those who responded, ‘I knew that’ and those who responded, “I wasn’t sure’ or ‘I didn’t know that’.

### Sociodemographic Variables

Participants were asked a range of sociodemographic questions. For age, ethnicity, region, and highest level of education, we grouped participants into categories where we hypothesised differences based on previous research findings (age, ethnicity, education level) or our knowledge of trans people in Aotearoa (region). For employment status, we grouped participants into those who were employed (full-time or part-time) and those who were unemployed (including students and retired people who weren’t in paid work). For free time spent with GBM, we asked participants to rate ‘how much of your free time is spent with gay, bisexual, takatāpui, or other men who have sex with men?’ and grouped participants into those who responded with ‘none’ or ‘a little’ versus ‘some’ or ‘a lot’. For participants’ financial situation, we asked, ‘how would you describe your money situation right now?’ and dichotomised responses into those who responded ‘comfortable, with extra’ or ‘enough, but not extra’ versus those who responded, ‘have to cut back’ and ‘cannot make ends meet’. To determine if participants had a disability, we asked participants, ‘do you have any long-term disability (lasting 6 months or more)?’.

### Statistical Analysis

We report the frequencies for all variables stratified by five gender groups: cis GBM, TW, TM, non-binary participants AMAB, and non-binary participants AFAB. For all non-sociodemographic variables, we conducted binary logistic regression tests to determine if there were any differences between cis GBM and each trans participant group. We report odds ratios (OR) adjusted for age, based on previous NZ research that trans populations tend to be younger (Stats NZ). We conducted all statistical analyses with STATA (Version 18.0).

## Results

Table 1 shows the demographic breakdown of participants by gender group. SPOTS 2022 recruited 3,838 participants, mainly cis GBM (90.8%, *n* = 3485), with smaller numbers of trans participants (9.2%, *n* = 353). Trans participants were mainly non-binary participants AMAB (3.8%, *n* = 144) and trans men (2.9%, *n* = 113), and there were lower proportions of non-binary participants AFAB (1.7%, *n* = 67) and trans women (0.8%, *n* = 29). Compared with cis GBM, a greater proportion of trans participants were younger, had lower rates of employment, had a tertiary education, and reported that they had to cut back financially or could not make ends meet. Compared to cis GBM (12.1%), higher proportions of trans participants reported a long-term disability, ranging from 34.3% for non-binary AMAB participants to 73.5% for non-binary AFAB participants. There appeared to be no consistent pattern to the ethnic distribution of participant groups, region or free time spent with gay men (Table 1).

**Table 1:** Socio-demographic characteristics of transgender and non-binary participants in the SPOTS survey 2022

Characteristic	Participant group									
	Cis GBM *N* = 3485		Trans women *N* = 29		Trans men *N* = 113		Non-binary AMAB *N* = 144		Non-binary AFAB *N* = 67	
	n	%	n	%	n	%	n	%	n	%
*Age group*										
<30	1072	39.5	16	59.3	81	81.8	77	70.6	40	81.6
30+	1640	60.5	11	40.7	18	18.2	32	29.4	9	18.4
*Ethnicity*										
Māori	418	12.1	8	28.6	15	13.3	36	25.4	18	26.9
Other	3026	87.9	20	71.4	98	86.7	106	74.7	49	73.1
*Highest education*										
Up to tertiary	1141	42.3	20	74.1	60	60.6	68	62.4	32	66.7
Tertiary	1559	57.7	7	25.9	39	39.4	41	37.6	16	33.3
*Region*										
Auckland or Wellington	1652	60.9	17	63.0	59	59.6	70	64.2	27	55.1
Rest of NZ	1059	39.1	10	37.0	40	40.4	39	35.8	22	44.9
*Employment status*										
Employed	2207	81.4	17	63.0	45	45.9	59	54.1	24	49.0
Not employed	505	18.6	10	37.0	53	54.1	50	45.9	25	51.0
*Free time spent with GBM*										
A lot/some	2311	66.9	12	41.4	92	81.4	103	73.1	56	83.6
A little/none	1143	33.1	17	58.6	21	18.6	38	26.9	11	16.4
*Money situation*										
Comfortable/enough	2326	86.2	16	59.3	74	75.5	76	69.7	26	54.2
Have to cut back/cannot make ends meet	374	13.9	11	40.7	24	24.5	33	30.3	22	45.8
*Long-term disability*										
Yes	326	12.1	12	46.2	46	46.5	37	34.3	36	73.5
No	2373	87.9	14	53.8	53	53.5	71	65.7	13	26.5

GBM = gay, bisexual and other men who have sex with men. AMAB = assigned male at birth. AFAB = assigned female at birth or sex assigned at birth not specified. NZ = New Zealand

Participants’ sexual partnering and HIV prevention behaviours are shown in Table 2. TM and all non-binary participants were less likely to have had sex with a man, and all trans participants were more than three times as likely to have had sex with a woman or with a non-binary person in the last six months compared to cis GBM. TW and non-binary AMAB participants were as likely as cis GBM to report two or more recent male sexual partners, whereas TM and non-binary participants AFAB were less likely than cis GBM to report multiple male partners. However, TW and non-binary AMAB participants were less likely than cis GBM to have a regular male partner. For HIV prevention behaviours in the last six months, TM and non-binary AFAB participants were less likely to have had any condomless anal intercourse with casual partners and less likely to have used PrEP in the last six months than cis GBM, whereas there was no statistically significant difference in the odds of TW and non-binary AMAB participants using PrEP. TW and non-binary AFAB participants were less likely to be willing to use the pill form of PrEP than cis GBM. TM had higher combination HIV prevention coverage with casual partners in the last six months than cis GBM.

**Table 2:** Sexual partnering and HIV prevention behaviours of transgender and non-binary participants in the SPOTS survey 2022

Behaviour	Participant group														
	Cis GBM *N* = 3485			Trans women *N* = 29			Trans men *N* = 113			Non-binary AMAB *N* = 144			Non-binary AFAB *N* = 67		
	n	%		n	%	OR (95% CI)	n	%	OR (95% CI)	n	%	OR (95% CI)	n	%	OR (95% CI)
*Sex with a man*															
6 m	3081	89.9	ref	26	89.7	1.27 (0.3–5.4)	85	75.2	**0.3 (0.2–0.4)**	108	77.7	**0.3 (0.2–0.5)**	50	75.8	**0.3 (0.2–0.6)**
6 m ago/never	345	10.1		3	10.3		28	24.8		31	22.3		16	24.2	
*Sex with a woman*															
6 m	275	8.0	ref	8	27.6	**4.9 (2.0–11.8)**	29	25.9	**4.4 (2.6–7.3)**	28	19.9	**3.5 (2.1–5.9)**	26	38.8	**8.0 (4.3–15.0)**
6 m ago/never	3160	92.0		21	72.4		83	74.1		113	80.1		41	61.2	
*Sex with a non-binary person*															
6 m	281	8.3	ref	13	48.2	**11.1 (5.0–24.6)**	29	26.4	**3.9 (2.4–6.3)**	62	43.1	**8.1 (5.3–12.2)**	29	43.3	**8.9 (4.9–16.1)**
6 m ago/never	3125	91.8		14	51.9		81	73.6		82	56.9		38	56.7	
*Number of male partners < 6 m*															
None or one	1375	40.5		10	34.5		73	64.6		61	43.9		41	62.1	
Two or more	2019	59.5	ref	19	65.5	1.4 (0.6–3.1)	40	35.4	**0.4 (0.3–0.6)**	78	56.1	0.8 (0.6–1.2)	25	37.9	**0.5 (0.3–0.9)**
*Current regular male partner*															
Yes	1706	51.8	ref	8	28.6	**0.3 (0.1–0.8)**	50	44.3	0.8 (0.5–1.2)	49	36.0	**0.5 (0.3–0.7)**	23	37.1	0.6 (0.3–1.0)
No	1589	48.2		20	71.4		63	55.8		87	64.0		39	62.9	
*Any condomless anal intercourse with casual partners < 6 m*															
Yes	1444	44.0	ref	9	32.1	0.7 (0.3–1.5)	17	15.5	**0.2 (0.1–0.4)**	60	43.8	0.9 (0.6–1.2)	11	16.9	**0.2 (0.1–0.5)**
No	1839	56.0		19	67.9		93	84.6		77	56.2		54	83.1	
*PrEP < 6 m* ^a^															
Yes	803	25.7	ref	4	14.8	0.5 (0.2–1.4)	8	7.4	**0.2 (0.1–0.5)**	22	16.9	0.6 (0.4–1.0)	6	10.2	**0.3 (0.1–0.8)**
No	2324	74.3		23	85.2		100	92.6		108	83.1		53	89.8	
*Willing to use PrEP pill* ^a^															
Yes	2307	78.2	ref	15	53.6	**0.3 (0.2–0.7)**	78	73.6	0.6 (0.4–1.0)	98	77.8	0.8 (0.5–1.3)	37	64.9	**0.5 (0.3–0.8)**
No	644	21.8		13	46.4		28	26.4		28	22.2		20	35.1	
*Combination HIV prevention coverage with casual partners* ^b^ *< 6 m*															
Yes	2605	83.2	ref	21	77.8	0.7 (0.3–1.7)	96	89.7	**1.9 (1.0–3.7)**	98	76.6	0.8 (0.5–1.3)	56	90.3	2.6 (0.9–7.4)
No	525	16.8		6	22.2		11	10.3		30	23.4		6	9.7	

GBM = gay, bisexual and other men who have sex with men. AMAB = assigned male at birth. AFAB = assigned female at birth or sex assigned at birth not specified. <6 m = in the last six months

^a^Not asked of participants living with diagnosed HIV. Includes daily and episodic pills, excludes participants only willing to use injectable PrEP. ^b^ Combination HIV prevention coverage defined as participants who: avoided sex or anal intercourse with casual male partners, were using PrEP, were living with diagnosed HIV with had an undetectable viral load, or used condoms consistently

Table 3 shows participants’ HIV and STI screening and self-reported diagnoses. No TW, TM or non-binary AFAB participant reported living with HIV. A small number (*n* = 3, or 2.3%) of non-binary participants AMAB had been diagnosed HIV positive. TM and non-binary participants were universally less likely to have tested for HIV in the last 12 months than cis GBM. There were no significant differences between trans participants and cis GBM in having a sexual health checkup in the last 12 months, and over half of all groups reported this. A similar proportion of participants reported an STI diagnosis in the last 12 months, with the exception of TM, who were less likely to have been diagnosed with an STI than cis GBM (Table 2).

**Table 3:** HIV testing and sexual health screening behaviours of transgender and non-binary participants in the SPOTS survey 2022

	Participant group														
	Cis GBM *N* = 3485			Trans women *N* = 29			Trans men *N* = 113			Non-binary AMAB *N* = 144			Non-binary AFAB *N* = 67		
	*n*	%		*n*	%	OR (95% CI)	*n*	%	OR (95% CI)	*n*	%	OR (95% CI)	*n*	%	OR (95% CI)
*HIV positive*															
Yes	151	4.9	ref	0	0.0	-^a^	0	0.0	-^a^	3	2.3	**0.7 (0.2–2.7)**	0	0.0	-^a^
No	2941	95.1		25	100.0		106	100.0		128	97.7		59	100.0	
*Tested for HIV < 12 m* ^b^															
Yes	1791	61.3	ref	12	48.0	0.5 (0.2–1.2)	39	36.8	**0.3 (0.2–0.5)**	65	51.2	**0.6 (0.4–0.8)**	24	40.7	**0.4 (0.2–0.7)**
No	1132	38.7		13	52.0		67	63.2		62	48.8		35	59.3	
*Sexual health checkup < 12 m*															
Yes	1878	61.8	ref	17	60.7	1.0 (0.5–2.2)	62	60.8	0.9 (0.6–1.3)	71	55.9	0.8 (0.5–1.1)	33	62.3	1.0 (0.6–2.0)
No	1159	38.2		11	39.3		40	39.2		56	44.1		20	37.7	
*Diagnosed with an STI < 12 m*															
Yes	521	17.4	ref	3	10.7	0.6 (0.2–1.9)	7	7.1	**0.4 (0.2–0.8)**	20	16.3	0.9 (0.6–1.5)	5	9.4	0.5 (0.2–1.2)
No	2468	82.6		25	89.3		92	92.9		103	83.7		48	90.6	

GBM = gay, bisexual and other men who have sex with men. AMAB = assigned male at birth. AFAB = assigned female at birth or sex assigned at birth not specified. <12 m = in the last 12 months

^a^Not calculated due to low numbers. ^b^Of those not living with diagnosed HIV

Table 4 shows participants’ healthcare experiences, substance use, and attitudes and knowledge about HIV prevention. TW, TM, and non-binary participants AFAB were more likely to report that their GP knew about their sexuality or their sexual behaviour with GBM than cis GBM. However, we also found that trans participants were more likely to have ever had a negative experience with a healthcare provider regarding their sexuality or sexual health needs than cis GBM. Trans participants were universally more likely to have ever injected drugs than cis GBM, but there were no differences in recent methamphetamine use.

**Table 4:** Healthcare experiences, drug use, sexual safety and attitudes and knowledge about HIV prevention of transgender and non-binary participants in the SPOTS survey 2022

	Participant group														
	Cis GBM *N* = 3485			Trans women *N* = 29			Trans men *N* = 113			Non-binary AMAB *N* = 144			Non-binary AFAB *N* = 67		
	*n*	%		*n*	%	OR (95% CI)	*n*	%	OR (95% CI)	*n*	%	OR (95% CI)	*n*	%	OR (95% CI)
*GP knows about your sexuality/sexual behaviour with GBM*															
Yes	1742	63.4	ref	22	81.5	**2.9 (1.1–7.7)**	68	69.4	**1.8 (1.2–2.9)**	58	53.7	0.8 (0.6–1.3)	35	71.4	**1.9 (1.0–3.7)**
No	1008	36.7		5	18.5		30	30.6		50	46.3		14	28.6	
*Negative experience with healthcare provider ever*															
Yes	506	18.5	ref	13	50.0	**4.5 (2.1–9.8)**	33	33.7	**2.5 (1.6–3.9)**	34	31.2	**2.2 (1.4–3.3)**	25	50.0	**4.8 (2.7–8.4)**
No	2227	81.5		13	50.0		65	66.3		75	68.8		25	50.0	
*Methamphetamine < 6 m*															
Yes	113	4.1	ref	2	7.7	2.0 (0.5–8.6)	2	2.0	0.6 (0.2–2.6)	6	5.5	1.7 (0.7–3.9)	2	4.1	1.3 (0.3–5.6)
No	2620	95.9		24	92.3		97	98.0		103	94.5		47	95.9	
*Injected drugs ever*															
Yes	96	3.5	ref	4	14.8	**5.5 (1.8–17.0)**	5	5.1	**2.8 (1.1–7.2)**	6	5.6	**2.6 (1.1–6.2)**	4	8.2	**4.8 (1.6–13.8)**
No	2636	96.5		23	85.2		94	95.0		102	94.4		45	91.8	
*“The sex I have is as safe as I want it to be”*															
Agree/strongly agree	2393	88.6	ref	21	80.8	0.6 (0.2–1.5)	81	82.7	0.6 (0.4–1.1)	90	84.9	0.7 (0.4–1.3)	35	72.9	**0.4 (0.2–0.7)**
Disagree/strongly disagree	308	11.4		5	19.2		17	17.4		16	15.1		13	27.1	
*Knowledge about undetectable HIV viral load*															
Knew that	2308	85.6	ref	20	76.9	0.5 (0.2–1.4)	79	80.6	0.6 (0.4–1.1)	88	82.2	0.7 (0.4–1.2)	40	81.6	0.7 (0.3–1.4)
Didn’t know/unsure	388	14.4		6	23.1		19	19.4		19	17.8		9	18.4	
*Knowledge about HIV PrEP*															
Knew that	2492	92.3	ref	22	84.6	0.5 (0.2–1.3)	82	84.5	**0.4 (0.2–0.8)**	91	85.1	**0.5 (0.3–0.8)**	43	87.8	0.6 (0.2–1.4)
Didn’t know/unsure	207	7.7		4	15.4		15	15.5		16	15.0		6	12.2	

GBM = gay, bisexual and other men who have sex with men. AMAB = assigned male at birth. AFAB = assigned female at birth or sex assigned at birth not specified. <12 m = in the last 12 months

For sexual safety, non-binary participants AFAB were less likely to agree that the sex they have is as safe as they want it to be than cis GBM. TM and non-binary participants AMAB were less likely to know that correct PrEP use can decrease the risk of acquiring HIV than cis GBM. However, there were no significant differences compared with cis GBM in trans participants’ knowledge that someone living with HIV who has a UVL cannot pass on HIV to their sexual partners.

## Discussion

In this large and diverse HIV behavioural surveillance study of trans people who have sex with GBM in NZ, we found that the sexual behaviours, HIV prevention and screening practices, and engagement with sexual health care varied across participant groups. In general, trans participants were less sexually active with men but more likely to report sex with women and non-binary partners in the last six months. Trans participants typically reported less HIV exposure risk via condomless anal intercourse with men, but also in some cases lower uptake of protective tools such as PrEP. STI screening and diagnosis appeared similar across all participants, but few trans participants reported living with HIV, although rates of recent HIV testing were also lower than cis GBM. These general patterns were however punctuated with exceptions for some trans groups. For example, non-binary AMAB participants reported some similarities to cis GBM in their sexual partnering, HIV prevalence and PrEP uptake, and TW reported potentially greater HIV exposure risks than other trans groups. Importantly, each trans subpopulation had a unique behavioural profile that was different in some respects from both cis GBM and other trans participants.

Our paper is among the first to report the micro-disaggregated experiences of trans participants eligible for a country’s national behavioural surveillance system and compare them with cis GBM traditionally sampled [25]. HIV control is improving among cis GBM in many countries, and officials are turning attention to under-served sub-populations in the goal to eliminate HIV transmission for all key populations by 2030 [36, 37]. Our insights may be useful for programme leads considering the expansion of HIV prevention and care services to be inclusive of trans populations, and who are interested in surveillance systems that can identify priority interventions and monitor progress. Our findings fill a research gap as there are limited studies exploring HIV prevention domains among trans people who have sex with cis GBM, especially trans men and non-binary people as we have done. Moreover, there is limited sexuality-focused research on trans people in NZ to inform practice and future research.

In our study, potential exposure to HIV was lower among trans participants who have sex with GBM, and this is reflected in lower HIV diagnoses, lower PrEP uptake for some trans participants and lower HIV testing rates. It may be that many trans individuals in NZ are therefore engaging in HIV care in ways that are proportionate to their risk. Our findings that no TW, TM and non-binary AFAB participants were living with diagnosed HIV is consistent with the low representation of these populations in HIV epidemiological surveillance in NZ [32]. Furthermore, NZ’s early adoption of policies supporting needle exchange and sex work law reform has kept HIV transmission low among people who use drugs and sex workers, reducing the influence of these behaviours as drivers of HIV risk for trans populations. Consequently, the pathways that contribute to high HIV incidence among trans people in other countries are far less pronounced in NZ. While all trans participants in our study had engaged in sex with GBM in the preceding five years, and were therefore linked to a sexual network with relatively higher HIV prevalence, most GBM in NZ report high HIV prevention engagement and low levels of undiagnosed HIV [34, 38]. These features may now indirectly protect trans and other populations during sex with GBM.

This interpretation should be considered alongside several important caveats. Trans participants in our study had relatively low levels of recent HIV testing, increasing the possibility that any new infections may be diagnosed later [39]. HIV surveillance systems may also under‑record trans status among newly diagnosed individuals [2]. In addition, self-reported drug injection in trans participants in our study was higher than among cis GBM. Although HIV risk in NZ from needle sharing is currently low [40], this is a potential future route of HIV transmission for all populations following the recent and ongoing HIV outbreak in Fiji [41], given the close connections between the two countries. In general, trans participants were younger, more likely to experience financial strain, and reported markedly higher rates of long‑term disability than cis GBM. These structural inequities are well‑established determinants of HIV vulnerability and could influence access to prevention technologies, and the ability to navigate healthcare systems.

This divergence suggests that barriers to HIV testing may be specific to HIV‑related services or shaped by perceptions of HIV risk that differ from those of cis GBM. Lower testing rates may also reflect discomfort with services perceived as not trans‑competent, or a mismatch between trans people’s sexual partnering patterns and the messaging or settings in which HIV testing is promoted.

Our study has implications for delivering health services. All individuals who report sex with GBM ought to be offered HIV testing and prevention options such as condoms and PrEP [36]. The lower PrEP uptake reported by trans participants in our study compared to cis GBM suggests that services need to better meet the needs of trans individuals at increased risk of exposure. It was encouraging that trans participants in SPOTS demonstrated higher disclosure of their sexuality and sexual behaviours to their GP than was true for cis GBM. However, trans participants were then more than twice as likely to report a negative experience in their lifetime relating to their sexual health care needs.

This paradox highlights the relational labour trans people undertake to access care, and the persistent shortcomings of healthcare environments in responding safely and competently. Negative experiences may deter engagement with HIV prevention services, contribute to lower HIV testing, and undermine trust in providers [42, 43]. It is important for healthcare providers to be aware of individual risk factors for their patients rather than making assumptions about their sex life (i.e., whether and who they are having sex with). NZ guidelines regarding gender affirming sexual health care support clinical discussions about HIV PrEP and doxy-PEP with trans individuals sexually matrixed with GBM [44], and should be adopted more widely in primary care. The lower prior knowledge about HIV PrEP among TM and non-binary AMAB participants in our sample, and the lower willingness to use PrEP pills among TW and non-binary AFAB participants, collectively indicates that trans individuals may feel that PrEP is not relevant to them. Reduced willingness could also reflect concerns about interactions between gender-affirming hormone therapy and PrEP [45], and that daily oral PrEP is the only recommended option for people on testosterone based gender affirming hormone therapy [44]. HIV prevention social marketing should ensure that the wide range of combination HIV prevention options are promoted effectively to trans individuals who are having sex with GBM.

Our findings also have implications for HIV behavioural surveillance programmes. First, whether to “lump” or “split” trans participants (i.e. analyse as a single group, or as distinct subgroups) [46]. For our cross-sectional analysis, we split non-cis GBM into four discrete categories of TW, TM, non-binary AMAB, and non-binary AFAB. This decision was informed by the desire for conceptual purity and because our large sample size (*n* = 3838) permitted it, while retaining minimum viable sample sizes for each subgroup [47]. We believe our findings support the value of splitting, as it respects participant-centred identities, and we found meaningful differences in the experiences of each group [48]. Splitting therefore can reveal health inequities among trans individuals that might otherwise be masked when trans groups are lumped together or simply dichotomised with cis GBM. Alternatively, lumping may be necessary with smaller samples, to avoid privileging sex assigned at birth over gender identity (e.g. for non-binary people), for pragmatic reasons such as statistical analysis– so long as this is conceptually coherent– or when the research question focuses on the experiences of all non-cis GBM, i.e. trans people navigating HIV prevention and care spaces not necessarily designed for them. Similar trade-offs have been noted in HIV research on ethnic inequities [49]. Splitting may also not be appropriate for education or health promotion. Ultimately, the choice whether to lump or split should reflect the research question, and other researchers have made different decisions [25].

Second, how might surveillance programmes report trends in behaviours over time for trans participants? Disaggregating trans participants as described above would allow progress in eliminating HIV prevention disparities to be monitored over time, so long as the subpopulation sample sizes were sufficiently powered to measure change. On the other hand, combining trans participants together with cis GBM would be more efficient analytically and for reporting. However, this approach risks further conflating and concealing improvements experienced by cis GBM, since changes in PrEP and testing uptake across successive data collection rounds would be diluted by combining multiple subpopulations, each with different levels of engagement (and targeting) in HIV prevention and care services. These considerations are further complicated where historical behavioural surveillance data for trans participants are limited, or where TM may have participated in previous rounds but could not be correctly identified without items on sex assigned at birth. More research is needed on how country-level surveillance systems are addressing these questions [50], and how data end users such as policymakers, public health authorities and service providers interpret and act on the data reported.

Third, trans individuals who have sex with GBM clearly view participating in HIV behavioural surveys as relevant. This mirrors a trend of greater visibility in official databases of historically minoritised communities [29] and greater willingness of rainbow populations to be counted, especially in health research [51]. Questionnaires ought to consider adding trans-relevant behavioural items, anatomically specific terms, and inclusive and respectful language [13]. At the same time, adaptations to “legacy” surveillance programmes need to keep items as consistent as possible to ensure comparisons over time remain valid [13, 25]. Achieving an appropriate balance between modernising measures to reflect contemporary gender terms and preserving continuity with earlier survey rounds is essential if surveillance data are to remain inclusive, analytically reliable, and useful for interpreting public health progress.

Fourth, inviting trans individuals into legacy surveillance systems requires an expansion of activities at all phases of the research process. As with cis GBM, research teams should consult trans community members in the design phase, ensure trans representation in study marketing, and consider specific audiences and strategies for data translation and dissemination. The SPOTS programme has already integrated many of these activities into the 2025 survey round [52]. Growing attention and emerging questions on social license in scientific research underscore that communities are reluctant to trust and participate in research unless research teams demonstrate transparency and show how their work will benefit community-defined priorities [53, 54]. Such work is heavily relational, and consequently also needs capability and funding.

There are several strengths to our study. SPOTS is one of the longest-running, largest and most diverse HIV behavioural surveillance programmes internationally. It is also the only NZ study on HIV prevention among trans women and non-binary people who have sex with GBM. The questionnaire included a wide range of behaviours relevant to understanding HIV risk that are modifiable by focused public health action. As this is an ongoing programme, future rounds can monitor changes in behaviours reported by trans participants in response to public health delivery, providing an accountability mechanism back to communities, an ability to assess whether services are meeting trans communities’ needs, and demonstrating the impact of public health investment in ways that uphold community trust and expectations. Future qualitative research would also develop our understanding of these findings by exploring how trans people interpret their reported risk and prevention behaviours, including how they assess their HIV risk, how different forms of sexual partnering shape that perception, and how these meanings reflect NZ’s distinct epidemiological and cultural contexts.

Our study also has limitations. SPOTS is a non-probability community sample, which means the findings are not generalisable to broader cis GBM and trans populations. We had a low number of trans participants, particularly trans women. Our findings apply only to trans participants who are sexual partners of GBM and are not representative of all trans populations. Survey items did not ask about the full range of possible genital contact configurations by or with trans participants. The gender of some sexual partners may have been misreported by participants. A small number (*n* = 11) of non-binary participants who preferred not to state their sex assigned at birth were classified as non-binary people AFAB; this may have been incorrect. Although we controlled for age in our analyses, we did not have statistical power to control for other potentially confounding variables, such as disability, education, or income. Some trans people, such as migrants or refugees, may have been less likely to participate due to English-language barriers or concerns that participation might influence their immigration status, despite the survey being anonymous. Other eligible people may not have heard about the survey, despite extensive recruitment efforts, particularly those who are older, transitioned a long time ago, or otherwise not engaged with LGBTQI+ organisations or communities, sexual health services, or sexual networking apps or porn sites used for recruitment. Although we believe the 2022 round improved the inclusivity of the SPOTS questionnaire and fieldwork, more rigorous efforts are possible which could have further increased participation by TNB people [13].

## Conclusion

This study provides the first detailed behavioural surveillance data on trans people who have sex with GBM in NZ, and demonstrates the value of disaggregating trans participants by gender and sex assigned at birth. Doing so revealed clear differences in sexual partnering, HIV prevention practices, and healthcare experiences that would have been obscured if trans people were treated as a single group or combined with cis GBM. Taken together, the findings suggest that HIV risk and vulnerability among trans people who have sex with GBM in NZ is shaped less by individual behaviours and more by structural conditions, sexual partnering patterns, and uneven access to affirming, competent healthcare.

## Data Availability

The datasets generated and/or analysed during the current study are not publicly available due to participant privacy, but are available from the corresponding author on reasonable request.
